# A systematic review of treatment options for post-prostatectomy incontinence

**DOI:** 10.1007/s00345-022-04146-5

**Published:** 2022-09-15

**Authors:** Alexander Canning, Nicholas Raison, Abdullatif Aydin, Samy Cheikh Youssef, Shamim Khan, Prokar Dasgupta, Kamran Ahmed

**Affiliations:** 1 Guy′s Kings and St Thomas′ School of Medical Education, King′s College London, London, UK; 2 Guy′s Hospital, King′s College London, London, UK

**Keywords:** PPI, Prostate, Prostatectomy, Urology, Incontinence, Post-prostatectomy

## Abstract

**Purpose:**

Urinary incontinence remains common in men after prostatectomy. Current guidance suggests early corrective surgery to those that are still incontinent after trying Pelvic Floor Muscle Therapy, however, other treatments are now available. This review aims to evaluate all currently available treatment options for men with post-prostatectomy incontinence (PPI).

**Methods:**

A search of MEDLINE and CENTRAL databases on 2/2/2021 produced 879 articles. Any study evaluating incontinence before and after a treatment protocol was eligible for inclusion. After screening, 17 randomized control trials were included, and pre-defined data points were collected. Due to heterogeneity, pooled analysis was not possible, and a descriptive synthesis was produced in accordance with PRISMA guidelines. Cochrane Risk of Bias (RoB) tool was used to evaluate all studies. The search protocol and methods for this study was registered on the PROSPERO database before the search began, ID:(CRD42021229749).

**Results:**

3/17(18%) of studies focussed on pharmacotherapy, 2/17(12%) on vibration therapies, 8/17(47%) on pelvic floor muscle therapy (PFMT), 3/17(18%) on electrical stimulation (ES), and 1/17 (6%) on extracorporeal magnetic innervation (ExMI) as their main intervention. The use of Duloxetine, Solifenacin, PFMT, ES, and ExMI all show effective reduction in incontinence in men suffering from PPI. No study in this review evaluated surgical managements for PPI.

**Conclusion:**

A large number of treatments are available for PPI using an array of different methods. For this reason, a variety of treatments could be considered before early invasive procedures, to prevent unnecessary surgery and its associated negative complications.

## Introduction

While 80% of incontinent men return to their baseline continence after prostatectomy without direct treatment [1], there is a cohort of those that suffer from refractory incontinence that does not resolve naturally. For those that remain incontinent, the resultant impact on their quality of life and patient autonomy can be hugely detrimental [2, 3]. The community follow-up in men with urinary storage symptoms has been estimated at £303 million per year in previous years, a substantial amount [4]. This review aims to address men after prostatectomy and evaluate the efficacy of all treatments for incontinence compared to control to recognize any changes in outcomes of incontinence, such as 24 h pad test or ICIQ scores.

Post-operatively, the European Association of Urology (EAU) and American Urological Association (AUA) guidelines both recommend Pelvic Floor Muscle Therapy (PFMT) as the first-line for post-prostatectomy incontinence (PPI) with a view of surgical treatments if PFMT fails.

As the current literature base for PPI treatment is limited [5], this review aims to evaluate the wide variety of treatment options that are available with the hope of integrating more options into clinical guidance.

## Methods

On 2/2/2021, MEDLINE and CENTRAL databases were searched using the following Boolean text filter for relevant terms:

("radical prostatectomy"OR"prostatectomy"OR"postprostatectomy") AND("incontinence"OR"urinary incontinence"OR"stress incontinence"OR"PPI").

Only studies originally published in English were eligible for inclusion. Using methodological filters, only randomized control trials (RCT) or prospective comparative studies were eligible for inclusion, published on any date before 2/2/21.

Studies were included into this review if; participants underwent radical prostatectomy, all participants suffered from urinary incontinence at the beginning of the study, and a specific treatment or combination of treatments was given prospectively, with follow-up.

Studies were excluded from this review if; participants also underwent previous surgery, including Transurethral resection of the prostate, radiotherapy, the study did not assess baseline continence, or included all men after prostatectomy.

The PRISMA framework for systematic reviews was referenced at every stage of this synthesis and consideration for associated guidance is found herein [6].

**Fig. 1:**
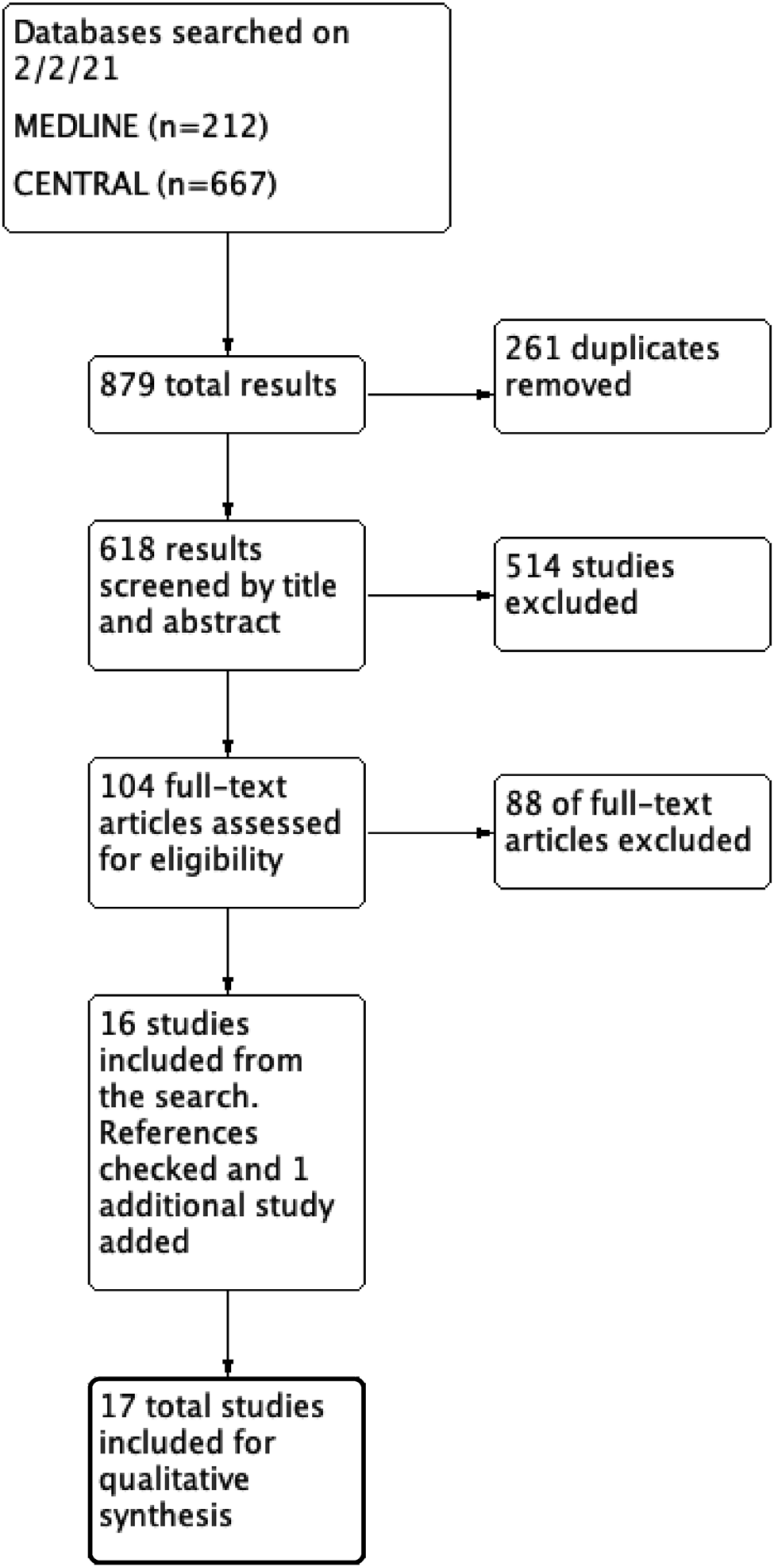
Flow diagram showing the process of study selection

After removal of duplicates, two reviewers independently screened the search for relevant studies based on their title and abstract. This group was then screened again based on the full-text article. If at any stage a full-text article was unavailable, or relevant information was missing, that study was excluded. Conflicts were discussed between the reviewers, with a third reviewer used to settle further debate. Figure 1 describes the inclusion process.

Data were extracted from included studies using a predefined data collection sheet containing; First author’s name, year and journal published, language, study design, method of randomisation and blinding, participant size per group, mean population data (such as age, BMI, and type of surgery), specific outcome measure and means with standard deviation of these measures for each group. As outcome measures vary within the literature, any quantitative measure of incontinence was included. Most commonly this included 24 h and 1 h pad tests, ICIQ scores, and incontinence diaries.

**Fig. 2:**
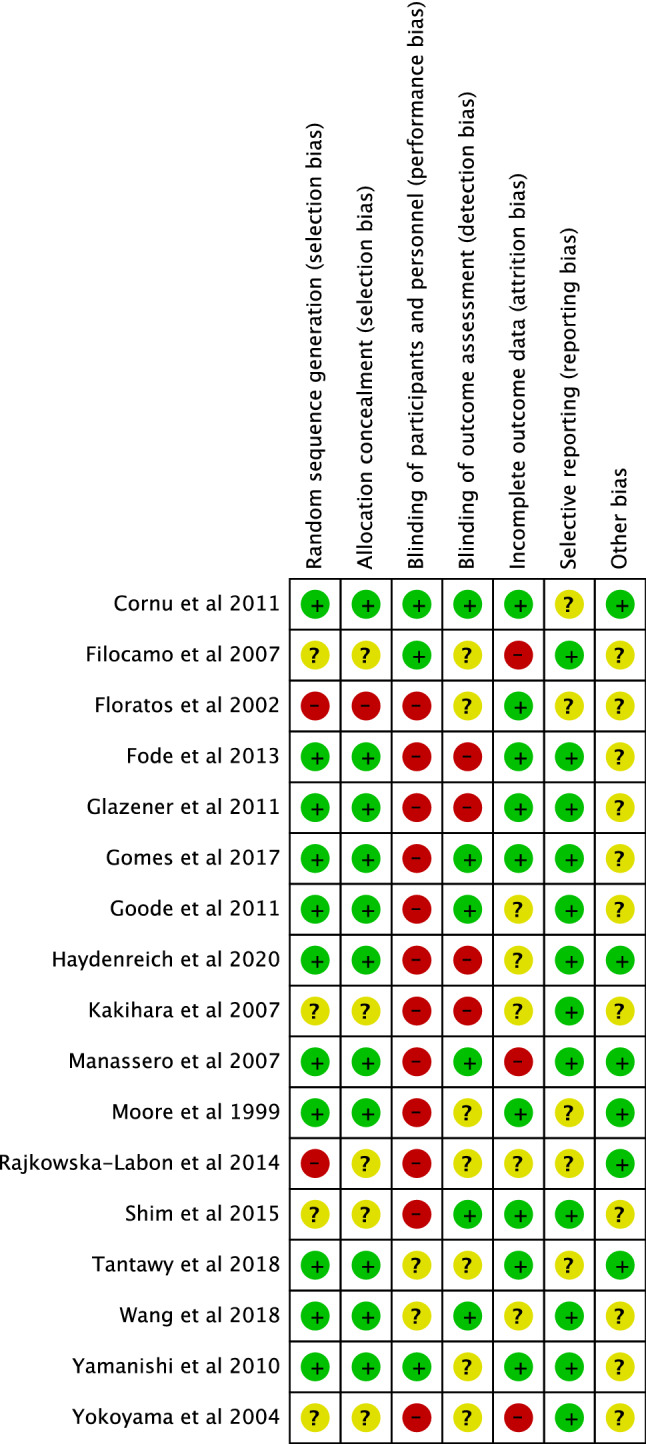
Risk of Bias assessment

Relevant data were input into RevMan 5 software for Risk of Bias assessment using the Cochrane risk of bias tools at the study level [7]. The following areas were assessed; Selection bias, Performance bias, Detection bias, Attrition bias, and Reporting bias, as seen in Fig. 2.

Of the included studies, 5 main categories of treatment protocol were defined and used to group studies. These groups were; Pharmacotherapy, Vibration therapy, PFMT, Electric Stimulation (ES), and Extracorporeal magnetic innervation(ExMI).

The search protocol and methods for this study were registered on the PROSPERO database before the search began, ID:(CRD42021229749).

## Results

In total from 879 search results, 17 studies were included with 1683 total included patients. Following grouping of similar studies, 3/17(18%) focussed on pharmacotherapy, 2/17(12%) on vibration therapies, 8/17(47%) on PFMT, 3/17(18%) on ES, and 1/17 (6%) on ExMI. More detailed information about the outcomes and conclusions of each study can be found in Table 1.

**Table 1:** A summary of included studies and associated outcomes, grouped by main intervention type. Population characteristics are included, along with the interventions and outcomes of each study, with standard deviations. (*n* = total number of included participants, SD = standard deviation, Int = Intervention group, Con = control group, RCT = randomized control trial, BID = twice daily) Where more than one intervention group was used, Int A and Int B are used to distinguish different interventions

Name, year, study type	Total participants, time after prostatectomy	Mean age(s) + SD	Arms of study	Data given
**Pharmacotherapy**				
*Cornu 2011, RCT [9]	*n* = 31, ≥ 1 year post-op	Int: 68.3(6.9), Con: 66.3(6.2)	Duloxetine 20 mg (BID) 7 d, then 40 mg BID for 67 d, and 20 mg BID for 14 d. (*n* = 16) versus placebo (*n* = 15)	% Change in incontinent episode frequency at 12 weeks (SD): Duloxetine: -52.2%(38.6) Placebo: + 19.5%(43.5) Mean difference: 71.2%, (*P* < 0.05)
*Filocamo 2007, RCT[10]	N = 102, ≥ 10 days after catheter removal	Int: 64.6, Con: 65.7	40 mg Duloxetine + PFMT (*n* = 50), versus placebo + PFMT (*n* = 52)	% Continence at week 16: Duloxetine: 78% Placebo: 52% (*P* = 0.007)
*Shim 2015, RCT [11]	*n* = 78, ≥ 3 weeks post-op	Int: 65.7, Con: 66.9	Solifenacin + midodrine (group 1, *n* = 39) versus midodrine alone (group 2, *n* = 39)	Difference in mean weight of daily pads after 4 months: Group 1: -52 g Group 2: -12 g (*P* = 0.005)
**Vibration Therapy**				
Fode & Sonksen 2013, RCT[12]	*n* = 31, ≥ 1 year post-op	Int: 67, Con: 67	Penile Vibratory stimulation (PVS) versus no treatment in a crossover RCT. (Early PVS *n* = 15, Late PVS *n* = 16)	No significant difference in change on pad test between groups at 6 weeks (*P* = 0.13)
*Tantawy et al. 2018, RCT[13]	*n* = 60, ≥ 6 months post-op	Int: 64.3(5), Con: 63.6(5.8)	Whole body vibration therapy + PME at home (group 1, *n* = 30) versus PME at home only (group 2, *n* = 30)	Whole body vibration showed a significantly greater reduction in 24-h pad test weight than control, (p < 0.05) at 2 months (90.5 g to 29.6 g vs 90.0 g to 52.7 g)
**Pelvic floor muscle therapy (PFMT)**				
Floratos et al. 2002, RCT [14]	*n* = 42, ≥ 1 week after catheter removal	Int: 63.1(4), Con:65.8(4.3)	PFMT with biofeedback + PME at home (*n* = 28) versus PFMT without feedback + PME at home (*n* = 14)	No significant difference on 1 h pad tests (Baseline, 1, 2, 3, 6 months) Feedback: 39 g, 18 g, 7 g, 4 g, 3 g Non-Feedback: 31 g, 11 g, 3 g, 1 g, 0 g (*P* > 0.05)
Glazener et al. 2011, RCT [15]	*n* = 411, ≥ 6 weeks post-op	Int: 62.4(5.8), Con: 62.3(5.6)	PFMT (*n* = 205) versus no treatment (*n* = 206)	Continence rate by ICIQ score at 12 months PFMT: 76% Control: 77% (*P* = 0.64)
*Gomes et al. 2017, RCT [16]	*n* = 104, ≥ 4 weeks post-op	Not stated	Pilates + PME at home (Int a, *n* = 34) versus PFMT with ES + PME at home (Int b, *n* = 35) versus no treatment (*n* = 35)	% of patients using 0 pads; Int A and Int B both superior to control (58.8% and 54.3% versus 25.7%) (*P* < 0.05) no difference between interventions *P* = 0.7. All after 10 weeks
*Goode et al. 2011, RCT [17]	*n* = 208, ≥ 1 year post-op	Int a: 66.3(7.5), Int b: 66.8(7.0) Con: 66.9(7.7)	PFMT (intervention a, *n* = 70) versus PFMT with biofeedback + ES at home (intervention b, *n* = 70) versus no treatment (*n* = 68)	Decrease in incontinent episodes after 12 months Int A and Int B both superior to control (*P* < 0.05), 55% and 51% versus 24% No difference between interventions *P* = 0.69
*Heydenreich et al. 2020, RCT [18]	*n* = 184, ≥ 4 weeks post-op	Int: 64.0(6.5), Con: 64.3(7.4)	PFMT with oscillating rod (*n* = 93) versus PFMT with relaxation therapy (*n* = 91)	Mean 1 h pad test (baseline, 3 week) Intervention: 22.6 g, 8.5 g Control: 23.0 g, 18.1 g After treatment, p = 0.008
*Manassero et al. 2007, RCT [19]	*n* = 94, ≥ 1 weeks after catheter removal	Int: 66.8(6.3), Con: 67.9(5.5)	PFMT + PME at home (*n* = 54) versus no treatment (*n* = 40)	lower incontinence rate with PFMT + PME versus control at 12 months: 52.5% versus 16.6% (*P* < 0.01)
Moore et al. 1999, RCT [20]	*n* = 58, ≥ 8 weeks post-op	Int a: 67.4, Int b: 65.7 Con: 66.8	PFMT (*n* = 18) versus PFMT + ES (*n* = 19) versus no treatment (*n* = 21)	no differences between groups on 24 h pad test from baseline to 24 weeks (*P* = 0.80)
*Rajkowska-Labon et al. 2014, RCT [21]	*n* = 81, “after prostatectomy”	Int a: 66.9(7.07), Int b: 68.8(6.59) Con: 68.3(6.49)	PFMT with biofeedback + PME at home (group Ia, *n* = 23) versus PFMT without feedback + PME at home (group Ib, *n* = 26) versus no treatment (group II, *n* = 32)	Improved continence with PFMT of either type (89% vs 11%, *P* < 0.005) Continence significantly improved without the use of biofeedback (92.3% vs 39.1%, *P* = 0.007). The length of study was until continence was achieved with a max of 12 months
**Electrical Stimulation (ES)**				
Kakihara et al. 2007, RCT [22]	*n* = 20, ≥ 6 weeks post-op	Total: 64.3(5.2)	PFMT with ES (*n* = 10) versus PFMT alone (*n* = 10)	Mean 1 h pad test (baseline, 12mo) no significant difference between treatment and control, *P* > 0.05. Both groups decreased mean 1 h pad test (28.0 g to 9.4 g and 9.0 g to 3.5 g, respectively) (*P* < 0.05)
*Wang et al. 2018, RCT [23]	*n* = 96, ≥ 1 month post-op	Not stated	Electrical pudendal nerve stimulation (EPNS) (*n* = 64) versus PFMT with biofeedback and ES + PME at home (*n* = 32)	Median ICIQ-UI SF score change after 8 weeks EPNS: 18 to 11 (*P* < 0.01) PFMT: 18 to 15 (*P* < 0.01) The difference between groups was 4 (*P* < 0.05)
*Yamanishi et al. 2010, RCT [24]	*n* = 47, ≥ 200 g daily incontinence ≥ 3 weeks post-op	Int: 65.4(5.6), Con: 68.0(5.6)	ES (*n* = 22) versus Sham ES (*n* = 25)	Time to continence: ES: 2.7 months Sham: 6.8 months (*P* < 0.05). Max follow-up of 12 months
**Extracorporeal Magnetic Innervation (ExMI)**				
*Yokoyama et al. 2004, RCT [25]	*n* = 36, incontinent 10–14 days post-op	Int a: 67.2(6.7), Int b: 68.2(4.9) Con: 66.2(7.6)	ES (*n* = 12) versus ExMI (*n* = 12) versus PFMT + PME at home (*n* = 12)	24 h pad test ExMI and ES – superior to control at 1- and 2-months *P* < 0.05 All groups insignificantly different from 3 months onwards to 6 months *P* > 0.05

*shows a significant result was found between groups

Outcome measures varied between studies, with the most common being 24 h pad test in 8/17(47%), ICIQ-SF questionnaire in 7/17(41%), and 1 h pad test in 6/17(35%). As the cohort of included studies was heterogeneous, a descriptive synthesis was performed following the Synthesis Without Meta-analysis guidelines (SWiM) [8].

Of the 15/17 studies that reported it, mean age ranged from 62.3 to 68.8. Of the 6/17 studies that reported it, mean BMI ranged from 24.6 to 30.1.

### Pharmacotherapy

Two studies (Cornu [9]and Filocamo [10]) conclude that Duloxetine is effective for PPI, for reduction to incontinent episodes (− 71.2%, *P* < 0.05) and 1 h pad-tests, respectively (+ 26% continence, *P* = 0.007). While Cornu [9] included those over 1-year post-prostatectomy, Filocamo [10] included those incontinent 10-days after catheter removal, showing efficacy of Duloxetine in a range of populations. All 3 pharmacotherapy studies showed improved continence compared to placebo.

Of note, 9 participants withdrew from the intervention group of Filocamo [10] due to side effects, compared to 1 in the placebo arm.

### Vibration therapy

Fode [12] showed mixed results in crossover RCT with patients over 1-year post-prostatectomy. The PVS first group showed significant reduction to 24 h pad-test at 6 weeks (− 33 g, *P* = 0.021), however, no significant difference to control of no treatment (*P* = 0.13). At 12 weeks after groups swapped protocol, there was an overall decrease to 24 h pad-test for the entire cohort (− 13.5 g, *P* = 0.004), however, PVS groups could not be shown to be superior at any point.

Tantawy [13] showed superior improvement with whole body vibration compared to PME only by 23.1 g at 2 months (*P* < 0.001).

### Pelvic floor muscle therapy (PFMT)

6 studies evaluated PFMT with conflicting results. Four studies (Rajkowska-Labon [21], Gomes [16], Goode [17] and Manassero [19]) with 487 patients showed significant improvements to continence in groups that underwent some form of PFMT. Conversely, two studies (Glazener [15] and Moore [20]) with 469 patients showed no significant improvements with PFMT compared to without.

Two studies (Floratos [14] and Goode [17]) both showed no significant improvement in the group that received biofeedback in addition to their PFMT. One study (Rajkowska-Labon [21]) went further, showing the protocol without biofeedback had a 53.2% higher continence rate than with biofeedback (*P* = 0.007).

### Electrical stimulation

3 studies investigated electrical stimulation (Kakihara [22], Wang [23], Yamanishi [24]), either alone or with PFMT. 2 studies (Wang [23] and Yamanishi [24]) found significantly improved continence in those receiving ES. Kakihara [22] showed no benefit of using ES with PFMT.

Wang [23] showed a decrease in ICIQ of 7 points in the EPNS group and Yamanishi [24] showed a mean reduction in time to continence of 4.11 months. Both conclusions support the use of ES.

### ExMI

Only one study involved ExMI (Yokoyama [25]) and found it effective in quickly reducing 24 h pad weight compared to PFMT, however, the benefit was no longer significant after 3 months. ExMI was equal to ES at every stage.

13/17 studies featured a high risk of bias in at least one domain as seen in Fig. 2. Due to the nature of interventions in all but the pharmacotherapy studies and one ES study, blinding was not possible. Performance bias was, therefore, found to be high risk in 12/17 studies.

Detection bias was the second most common field to be identified as high risk, as in 4 studies (Fode [12], Glazener [15], Heydenreich [18], and Kakihara [22]). This was due in 2 cases to the fact that unblinded participants self-reported their symptoms. In the other two cases, the outcome assessor was aware of the patient’s protocol.

## Discussion

Duloxetine increases the neural tone of the urethral sphincter, preventing incontinent episodes [26]. Efficacy has been seen in women with stress or mixed incontinence [27], however, the pathophysiology is believed to be different in men [28]. Table 1 shows efficacy in reducing incontinent episodes by 71.2% (*P* > 0.001) even 1-year after prostatectomy (as in Cornu [9]) and should, therefore, be considered a viable option to treat PPI in patients that can tolerate the side effects or who are unfit for surgery. The side effects of Duloxetine include nausea, dry mouth, and insomnia, [29] which are all barriers to use. These side effects are, however, limited to the early period and duloxetine can be used successfully with perseverance [30], although data for this are limited and out of the scope of this review.

The use of Solifenacin by Shim [11] showed a more significant decrease in men’s pad weight compared to control (− 39.8 g, *P* = 0.005), suggesting that anticholinergics may also be an effective mechanism for treating PPI. The use of Solifenacin was previously only discussed in the context of standalone urge incontinence [31], however, use in the PPI population might be effective. These results also report an increase to bladder capacity in the Solifenacin group, ultimately leading to improvement of overactive bladder symptoms and resolution of incontinence.

Of the two vibration-based studies, only that of Whole-Body vibration Tantawy [13] showed effective results in decreasing mean 24 h pad test weights by 23.1 g more in the group that received whole body vibration (*P* < 0.001). It is believed to function by strengthening muscles involved in continence [32]. PFMT also works to increase muscle strength and may achieve the same results as whole-body vibration devices without the associated costs and learning curve for the patient.

Penile vibration is believed to work by pudendal nerve sensitisation to facilitate contraction of the pelvic floor [33, 34]. One study (Fode [12]), showed limited efficacy in reducing 24-h pad weight, with no significant difference in pad test by 6 weeks (*P* = 0.013), supporting the theory that vibration therapy is only effective when the end result is pelvic floor strengthening.

As the current mainstay of first-line treatment, recommended by EAU and AUA guidelines [30], a majority (13/17) of the studies involved PFMT, with 8/17 directly targeting it. This review concludes that PFMT is often effective in PPI, either alone or with adjuncts. While two studies (Glazener [15] and Moore [20]) show insignificant results with PFMT compared to control (*P* = 0.64 and *P* = 0.80, respectively) a larger number of recent studies support its use. As the complications and side effects of PFMT are limited, there is little reason not to use PFMT.

The use of biofeedback is ineffective in men with PPI as in Floratos et al. ([14]) despite acceptance in the literature for use in stress incontinence [35]. It may be that the pathophysiology of PPI involves different mechanisms to that of stress incontinence which render biofeedback ineffective.

Pilates showed equal efficacy to PFMT in the amount of men using 0 pads (58.8% vs 54.3%, *P* = 0.7) in one study (Gomes [16]), again suggesting the underlying effect of increased pelvic and abdominal muscle tone is an effective way to improve PPI.

Two studies conclude the efficacy of electrostimulation to decrease incontinence for decreasing median ICIQ score (*P* < 0.05) and reducing time to continence (2.7 vs 6.8 months, *P* < 0.001) as in Wang et al. [23] and Yaminishi et al. [24], respectively. Only one study (Kakihara [22]) showed no significance of ES, however, the small sample size (*n* = 20) and very high risk of biases reduce the reliability of results.

The believed mechanism of ES is contraction and strengthening of the pelvic floor [36–38] which is both safe and effective. ES can be used by educated patients without the need for physiotherapists, which gives the opportunity for increased autonomy and lower staff involvement compared to ExMI, PFMT, or whole-body vibration therapy.

The mechanism of ExMI is suggested to be similar to that of ES [39]. No difference between ExMI and ES has been found in this review, with both showing quicker time to continence compared to PFMT and respective benefit.

Urethral bulking agents, sling procedures or artificial urinary sphincter (AUS) devices are often regarded as the definitive treatment when PFMT fails to improve a patient’s symptoms. This is reflected in the EAU and AUA guidelines which suggest surgical management as early as 6 months post-prostatectomy if conservative measures fail [30, 40]. This review, however, did not include any study involving surgical procedures. This could indicate a lack of high-level evidence for surgical measures, however, it could also be due to the difficult nature of carrying out RCTs with surgical procedures.

General surgical risks are associated with any invasive procedure, and this review highlights how other treatment options can be effective even years after prostatectomy. Devices such as AUS also decrease in efficacy over time, requiring revision surgery or replacement. Many treatments evaluated in this review show promising results, however, as the greatest length of follow-up of any study was only 12 months, it is not possible to compare the long-term efficacy against surgical procedures.

Due to variations in outcome measure and continence definition, the included studies in this review were significantly heterogeneous. The length of time after prostatectomy for study inclusion was another varying factor. The involvement of post-operative inflammation is known to contribute to urinary symptoms in the early postoperative period [41] and alleviates with time. Time after prostatectomy could, therefore, alter how receptive patients are to different treatment methods based on their healing.

Due to the nature of the treatments being investigated, risk of performance bias was unavoidably high as blinding was not always possible. Detection bias, however, can be avoided in future studies by successfully blinding the outcome assessor.

Overall, the quality and number of studies investigating this issue is limited, and even the studies that were included were of limited size. Larger and higher quality investigations of treatments for PPI are, therefore, warranted in future.

Of the included studies in this review, inconsistencies between definitions of incontinence and outcome measures have made meta-analysis impossible and direct comparison difficult. Universal standardisation of these definitions and outcome measures would make pooled analysis possible and would give a larger knowledge base to formulate future guidance. The invention and implementation of standardized questionnaires has aided in this; however, no overarching definition of even incontinence has been agreed.

## Conclusions

The use of Duloxetine, Solifenacin, PFMT, ES, and ExMI all show efficacy in reducing incontinent symptoms in men with PPI. Despite common use and acceptance in guidelines, no study included into this review evaluated surgical managements. This indicates a gap for high quality studies of surgical methods to validate their use. With such a wide variety of safe and effective treatment options available, a change of treatment or the use of adjunct therapies could be considered before surgery to avoid unnecessary interventions, complications and reoperations once surgical devices eventually fail over time.

## Synthesis without meta-analysis

To supplement the PRISMA guidelines [6] used to carry out this review, each of the following sections of the SWiM guidelines have been addressed in previous sections as listed.

**Table:**

SWiM reporting item	Area of manuscript where item is reported
1 – Grouping studies for synthesis	Methods
2 – Describe the standardized metric and transformation methods used	Methods
3 – Describe the synthesis methods	Methods
4 – Criteria used to prioritize results for summary and synthesis	Methods
5 – Investigation of heterogeneity in reported effects	Methods, Discussion
6 – Certainty of evidence	Figure 2, Discussion
7 – Data presentation methods	Results, Table 1
8 – Reporting results	Results, Table 1
9 – Limitations of the synthesis	Discussion, Conclusion
